# Technical assessment of whole body angiography and cardiac function within a single MRI examination

**DOI:** 10.1016/j.crad.2015.02.003

**Published:** 2015-06

**Authors:** S.J. Gandy, M. Lambert, J.J.F. Belch, I.D. Cavin, E. Crowe, R. Littleford, J.A. Macfarlane, S.Z. Matthew, P. Martin, R.S. Nicholas, A.D. Struthers, F. Sullivan, S.A. Waugh, R.D. White, J.R. Weir-McCall, J.G. Houston

**Affiliations:** a NHS Tayside Clinical Radiology, Ninewells Hospital, Dundee DD1 9SY, UK; b NHS Tayside Medical Physics, Ninewells Hospital, Dundee DD1 9SY, UK; c Medical Research Institute, College of Medicine, University of Dundee, Dundee DD1 9SY, UK; d Department of Family and Community Medicine, University of Toronto, Toronto, Canada; e Department of Research and Innovation, North York General Hospital, Toronto, Canada; f Department of Clinical Radiology, University Hospital of Wales, Cardiff CF14 4XW, UK

## Abstract

**Aim:**

To evaluate a combined protocol for simultaneous cardiac MRI (CMR) and contrast-enhanced (CE) whole-body MR angiography (WB-MRA) techniques within a single examination.

**Materials and methods:**

Asymptomatic volunteers (*n* = 48) with low-moderate risk of cardiovascular disease (CVD) were recruited. The protocol was divided into four sections: (1) CMR of left ventricle (LV) structure and function; (2) CE-MRA of the head, neck, and thorax followed by the distal lower limbs; (3) CMR LV “late gadolinium enhancement” assessment; and (4) CE-MRA of the abdomen and pelvis followed by the proximal lower limbs. Multiple observers undertook the image analysis.

**Results:**

For CMR, the mean ejection fraction (EF) was 67.3 ± 4.8% and mean left ventricular mass (LVM) was 100.3 ± 22.8 g. The intra-observer repeatability for EF ranged from 2.1–4.7% and from 9–12 g for LVM. Interobserver repeatability was 8.1% for EF and 19.1 g for LVM. No LV delayed myocardial enhancement was observed. For WB-MRA, some degree of luminal narrowing or stenosis was seen at 3.6% of the vessel segments (involving *n* = 29 of 48 volunteers) and interobserver radiological opinion was consistent in 96.7% of 1488 vessel segments assessed.

**Conclusion:**

Combined assessment of WB-MRA and CMR can be undertaken within a single examination on a clinical MRI system. The associated analysis techniques are repeatable and may be suitable for larger-scale cardiovascular MRI studies.

## Highlights

• We report the use of whole body MR angiography and cardiac MR as a single examination.
• Healthy volunteers with elevated cardiovascular disease risk were scanned.
• Vessel segments and cardiac function were assessed by Radiologists and Physicists respectively.
• The protocol took an average of 51 minutes to complete, and analyses were repeatable.
• This combined cardiovascular MRI protocol will be used for better targeting of future interventions.

## Introduction

Cardiovascular disease (CVD) accounts for a significant burden of mortality and morbidity in developed societies. Although the majority of CVD deaths are from coronary heart disease (CHD) or stroke, the disease is often spread across all vascular territories and may present elsewhere first, e.g., in the arteries of the legs. Disease in more than one vascular bed (especially when involving the peripheral arteries) is known to have a cumulative effect on worsening prognosis, so early detection and stratification of the whole-body burden of CVD (e.g., identifying those at highest risk of sudden death through CHD or stroke) is desirable. Primary prevention of CVD events is effective, but targeting suitable treatment, such as protective drug therapy and/or procedural interventions, to those most likely to benefit remains a challenge. Current decisions are based on estimated CVD risk, but risk scores do not have good external validity, and a significant number of events occur in those deemed to be at low or intermediate risk. Therefore, a method to screen for “pre-clinical” cardiovascular disease may help to improve how primary prevention is targeted.

Cardiac MRI (CMR) has developed into the imaging standard for evaluating cardiac left ventricular (LV) structure and function.^1^, ^2^ Numerous studies have defined normal ranges for LV parameters^3^, ^4^ and these have been stratified by demographics such as age,^5^ gender,^6^ and ethnicity.^7^ However, cardiac structure and function is only deemed to represent part of the solution towards a more comprehensive cardiovascular MRI assessment, where further desired information would be provided by arterial luminal imaging of the vascular tree.

Recent advances in MRI hardware such as radiofrequency (RF) coil connectivity have resulted in the emergence of the whole-body MR angiography (WB-MRA) technique,^8^, ^9^ which can potentially add to a more comprehensive whole-body cardiovascular assessment. The WB-MRA examination can be performed using a “stepping table” approach^10^ or by the use of continuously moving table methods.^11^ WB-MRA can be performed on 1.5^12^ or 3 T^13^ machines, but the use of a 3 T MRI system is deemed to present an advantage^14^ by virtue of the additional signal available that can be traded-off for improved in-plane resolution or faster scan times. Refinement work to the WB-MRA technique at 3 T has previously been reported and this has included the optimization of single injection strategies,^15^ double injection strategies,^16^ and contrast medium dose optimization.^17^ The use of CMR has also been validated at 3 T, where electrocardiogram (ECG)-gated two-dimensional (2D) segmented cine steady-state free precession (SSFP) techniques can be implemented and compare favourably with data acquired at 1.5 T.^18^

For data analysis, quantitative methods for 3 T CMR are widely described^19^, ^20^ and LV segmentation can be performed using a host of commercially available software packages. However, WB-MRA is more suited to qualitative approaches where scoring systems can report regional^21^ or total^22^ indices of CVD, which can then be correlated with symptoms in patients.^23^

The present study was undertaken to incorporate CMR and WB-MRA into a single 3 T “hybrid” clinical protocol capable of acquiring images of the vascular tree together with assessment of heart structure, function, and late gadolinium enhancement (LGE) within a single examination. The protocol consists of four sections, each made up of sequences (that are all widely available on commercial MRI systems) as follows: (1): CMR of LV structure and function; (2) contrast-enhanced (CE)-MRA of the head/neck/thorax and the distal lower limbs (contrast medium injection 1); (3) CMR of LGE; and (4) CE-MRA of the abdomen/pelvis (contrast medium injection 2).

To date, no work has been reported looking at the role of this combined protocol in a low or intermediate risk cohort, nor to assess the technical reproducibility of the study in such a population. The primary objectives of the study were therefore: (1) to implement this MRI protocol on a cohort of asymptomatic volunteers with known risk of CVD but with no previous clinical diagnosis; and (2) to evaluate data analysis strategies for CMR and WB-MRA with input from multiple observers in order to establish the intra- and interobserver repeatability. WB-MRA combined with CMR and LGE represents an attractive proposal for cardiovascular work given its systemic assessment of the heart and vascular tree. The WB-MRA technique on its own is known to correlate better with future cardiovascular risk than current scoring mechanisms in a high-risk population.^12^

## Materials and methods

Following local ethical committee approval, *n* = 48 volunteers (17 men, 31 women, mean age 54 years, range 41–71 years) were recruited after providing informed consent. Inclusion criteria were as follows: (1) >40 years; (2) free from CVD or other indication for statin therapy as recommended by the Scottish Intercollegiate Guidelines Network (SIGN) report 97 (www.sign.ac.uk) published in February 2007, and (3) had a serum B-type natriuretic peptide (BNP) level greater than their gender-specific median indicating non-specific stress on the cardiovascular system. Exclusion criteria included: (1) pregnancy; (2) known primary muscle disease; (3) known atherosclerotic disease, including unstable angina, previous myocardial infarction, peripheral arterial disease, amputation, revascularization, hypertension, heart failure, or cerebrovascular event; (4) known diabetes; (5) active liver disease; (6) other known illness or contraindication to MRI; (7) participation in a clinical trial; (8) inability to give informed consent; (9) known alcohol abuse; and (10) blood pressure of greater than 145/95 mmHg.

**Figure 1:**
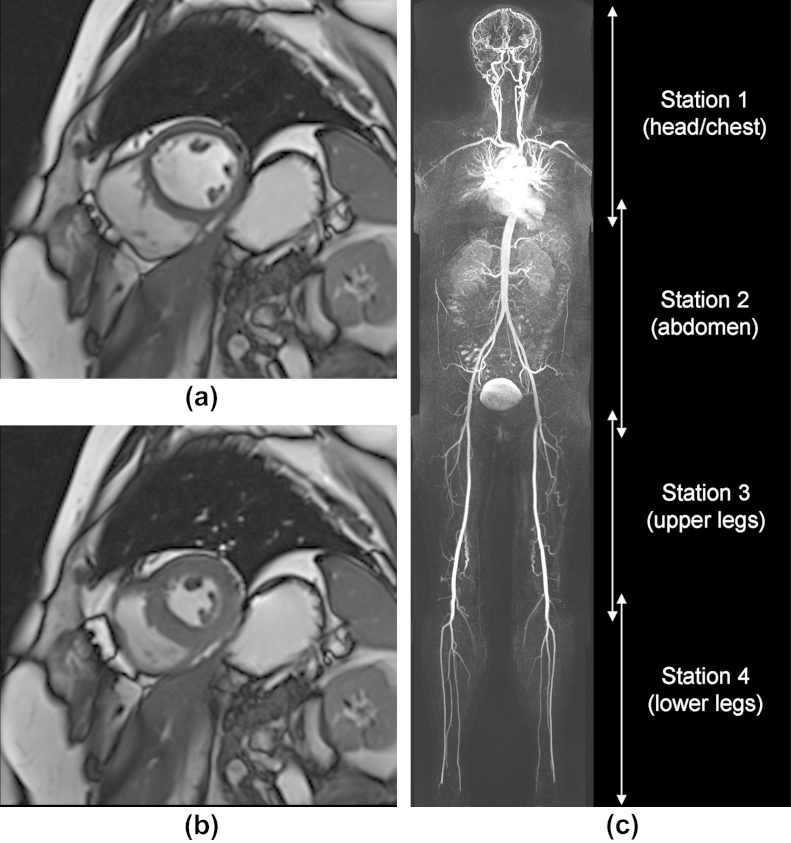
Example CMR short axis data (a) at end-diastole, (b) at end-systole, and (c) WB-MRA coronal plane MIP dataset acquired from a 50-year-old female patient with normal vascular segments. For WB-MRA, station 1 and 4 images are acquired after the first injection of contrast agent. Station 2 and 3 images are acquired after the second injection of contrast agent. Overlap between stations is at least 75 mm; however, care must be taken with interpretation of vessels at the station boundaries, as variable contrast medium in these areas has the potential to mimic a stenosis.

Imaging was performed (head-first, supine orientation) using a 3 T (102 × 32) Magnetom Trio Scanner (Siemens, Erlangen, Germany) with a coil combination using head (12 elements), neck (four elements), body (two coils of six elements each), spine (up to 24 elements), and peripheral angiography (16 elements) RF coils. Preliminary three-plane “localizer” images were acquired for WB-MRA via the use of 500 mm field-of-view (FOV) gradient echo fast low-angle shot (FLASH) sequences covering the anatomy from head to foot. Localizer images were positioned with an “overlap” between each FOV of at least 75 mm but adjustable according to patient height (Fig 1c). As part of the preparation phase, localizer TurboFLASH images were also acquired of the heart in the two-chamber (2ch), four-chamber (4ch) and short axis (SA) orientations.

### Protocol section 1: cine imaging and CMR of LV function

ECG-gated segmented breath-hold cine TrueFISP images were acquired in the LV 4ch and 2ch orientations. Following this, a stack of short axis images were acquired from the atrio-ventricular ring to the LV apex using 2D ECG-gated breath-hold segmented cine TrueFISP sequence with retrospective gating (Table 1).

### Protocol section 2: WB-MRA — stations 1 and 4

Unenhanced CE-MRA “mask” data were acquired for each station using a 3D TurboFLASH sequence, and the scanner table was preset to move at a rate of 50 cm/s. A 10 ml “standard dose” of 0.05 mmol/ml gadoteric acid (Dotarem^®^, Guerbet, France) followed by 20 ml saline flush was delivered at the left or right antecubital fossa using a Spectris Solaris power injector (MedRad, Pittsburgh, PA, USA) at a rate of 1.5 ml/s. Timing was controlled by a coronal 2D Care Bolus acquisition (MR fluoroscopy), and the contrast-enhanced acquisition for station 1 commenced when contrast agent arrival was noted at the top of the aortic arch. Post-contrast data for station 4 were acquired immediately after completion of station 1, and these were acquired three times consecutively to account for variable arterial transit times.

### Protocol section 3: CMR of myocardial viability with phase-sensitive inversion recovery (PSIR)

An ECG-gated segmented breath-hold 2D inversion-recovery prepared CINE TrueFISP “TI-Scout” sequence was implemented (in a central short-axis position) 8–10 min after initial contrast medium injection in order to identify the null point inversion time (TI) for the myocardium. Subsequently, at a mean of 11 min post-contrast medium administration (range 9–16 minutes) a short-axis stack of ECG-gated segmented 2D PSIR images were acquired in order to highlight any LGE within the myocardium. The mean TI used was 376 ms (range 300–450 ms).

### Protocol section 4: WB-MRA — stations 2 and 3

Unenhanced 3D Turbo-FLASH “mask” data were acquired for each station (Table 1). The second contrast agent dose was delivered the same as before but this time the “standard dose” was 15 ml, infused at 1.5 ml/s followed by a 20 ml saline flush. Timing was again controlled by 2D Care Bolus (coronal plane abdominal aorta), and post-contrast scans for station 2 were triggered when the bolus could be seen arriving in the abdominal aorta. Post-contrast data for station 3 were acquired immediately after completion of the station 2 sequence. The average time between first and second contrast agent injections was 19 min (range 15–34 min).

Finally, the pre- and post-contrast WB-MRA data were subtracted and the resulting images were stitched using a multi-modality work platform (MMWP; Composing, Siemens, Erlangen, Germany). Multiplanar reconstructions (MPR) and maximum intensity projections (MIP) were also generated for further radiological interpretation.

### Image analysis

For CMR, all datasets (*n* = 48) were analysed once by each of a team of four experienced medical physics observers in order to assess interobserver variation. Additionally the datasets were divided into four groups of *n* = 12, and one group was assigned to each medical physics observer for further segmentation, resulting in four independent assessments of intra-observer variation. For WB-MRA, all datasets were analysed once by each of a team of four radiologist observers in order to derive interobserver variation. Again the datasets were divided into four groups of *n* = 12, and one group was assigned to each radiologist observer for further segmentation, resulting in four independent assessments of intra-observer variation. Dataset randomization was performed by an independent support statistician (P.R.). Repeat analysis was separated by at least 1 month in order to eliminate “learning effects” by observers.

CMR images were analysed using commercial software (Argus, Siemens Multi-modality Work Platform, version VB 15). Region of interest (ROI) contours were placed around endocardial and epicardial LV borders on all CMR image sections at end-diastole and end-systole that contained 50% or more full-thickness myocardium. Quantitative measurements of EF and LVM (at end-diastole) were derived. Papillary muscles were included in the LVM if indistinguishable from the myocardial wall, but otherwise assigned to the left ventricular blood pool.

WB-MRA analysis was performed using a diagnostic PACS radiological workstation (Carestream PACS Client Suite Version 10.1 sp1, Rochester, NY, USA) on the post-contrast TurboFLASH source images, with MIP and MPR images for further interpretation. Analysis was performed on 31 arterial segments per dataset (Table 2). Radiological stenosis grading was performed by a visual examination of each vessel segment, and stenoses (if present) were recorded as a percentage of luminal diameter loss relative to a distal healthy segment. If more than one stenosis was present the most severe was assessed. The subdivision of longer arteries into smaller segments was performed to ensure that the presence of multiple pathologies affecting any particular arterial segment could be captured. A grading scale of 0–4 was applied to each arterial segment as follows: grade 0 = healthy segment, grade 1 = 1–50% stenosis, grade 2 = 51–70% stenosis, grade 3 = 71–99% stenosis, and grade 4 = occlusion. Non-diagnostic segments were recorded as “non-interpretable”. Finally, the vessel scores were summated on a per-volunteer basis to form a total atheroma burden score for each volunteer, and from this a standardized atheroma score (SAS) was derived using equation (1), which expresses a generalized atheroma burden severity across the body as a percentage (n = number of segments).

(1) $$SAS=\left[\left(\frac{\sum score}{n}\right)\times \frac{1}{4}\right]\times 100$$

### Statistical analysis

For the CMR LV function data, intra- and interobserver coefficients of repeatability (CoR) for EF and LVM were calculated as 2.77×(S_w_)^0.5^, where Sw is the within-subject standard deviation. One-way analysis of variance (ANOVA) was implemented (with 95% confidence limits) in order to highlight any significant differences between the means of the EF and LVM parameters both within and between observers. A minor variation to the standard Bland–Altman plot was used to illustrate interobserver variation for EF and LVM.^24^ For the WB-MRA data, agreement in stenosis grading was calculated using Fleiss' kappa statistic (*k*) for interobserver measures. Variability in whole-body atheroma scoring was assessed using the Kruskal–Wallis test for multiple observations and the Wilcoxon signed rank test was used to test for observational pairs of data. Statistical analysis was performed using IBM SPSS, version 21 (Chicago, IL, USA).

## Results

**Figure 2:**
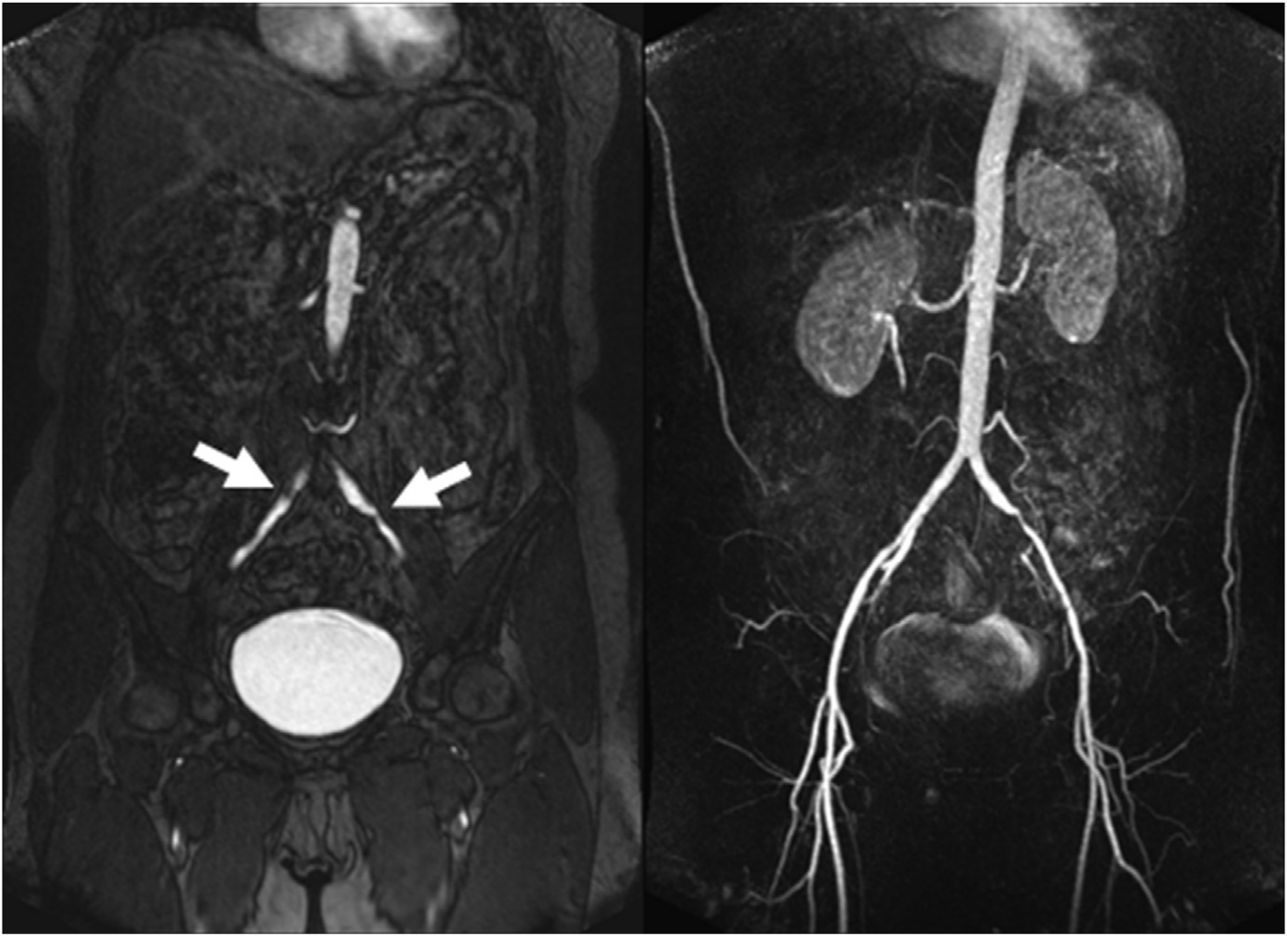
Example images highlighting stenoses (arrowed) in common iliac arteries on (left) a source image, and (right) a MIP image of the abdominal station of a 60-year-old woman. Of particular interest is the fact that the source images have been useful on this occasion for identifying pathology in the right iliac artery, which is not noted on the MIP reconstruction.

The CMR and WB-MRA datasets were acquired successfully and provided analysable data for all sequences and arterial segments, although one WB-MRA arterial segment (out of 1488) was deemed non-interpretable and excluded from further analysis. An example image dataset highlighting CMR (LV short axis) and the corresponding WB-MRA is illustrated in Fig 1, and examples of WB-MRA highlighting vascular disease are illustrated in Fig 2. The mean examination time was 51 min, although this was variable (standard deviation ± 10 min) and depended upon the patient size and the need for extra shimming steps in some cases.

For CMR, mean values (derived by *n* = 4 medical physics observers) for EF ranged from 65.3 ± 5.1% to 71.1 ± 4.7%, with a consensus mean of 67.3 ± 4.8% (for *n* = 48 patients). For LVM, mean values ranged from 92.8 ± 22.1 g to 105.3 ± 22 g, with a consensus mean of 100.3 ± 22.8 g (when normalized to body surface area,^25^ the mean LVM index values ranged from 49.4 ± 9.3 g/m^2^ to 56.1 ± 8.7 g/m^2^ with a consensus mean of 53.3 ± 9.1 g/m^2^). There were no cases of myocardial LGE identified by the radiologist observers.

**Figure 3:**
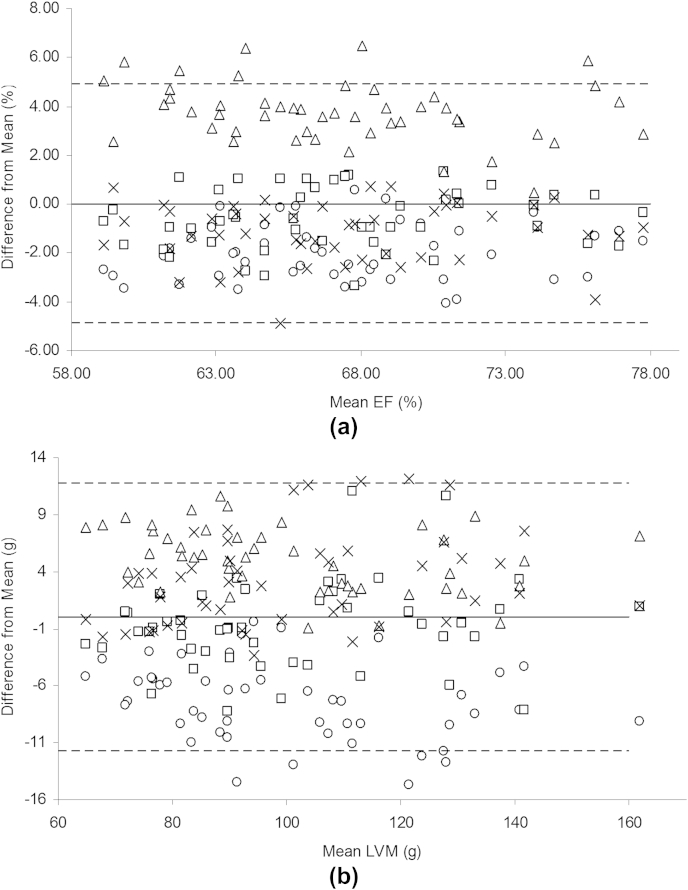
Interobserver data for EF (%) and LVM (g). Circles, observer 1; squares, observer 2; triangles, observer 3; and crosses, observer 4. The hashed lines represent two standard deviations from the mean difference between observers.

Data plots for multiple observers are presented in Fig 3, highlighting the variation in interobserver repeatability for EF and LVM. For EF, observer 3 tended to generate values that were larger than those from the other observers (*p* < 0.05). However, when EF data derived by the remaining observers were evaluated, there was no statistical difference detected between their means. The single-measure interobserver CoR for EF was 8.1%, and for each individual observer the test-retest intra-observer CoR for EF was 4.7% (observer 1), 2.1% (observer 2), 2.7% (observer 3) and 3% (observer 4). For LVM, observer 1 tended to generate values that were smaller than those from the other observers (*p* < 0.05). Again, when LVM data derived by the remaining observers were considered there was no statistical significance detected between their means. The single-measure interobserver CoR for LVM was 19.1 g, and for each individual observer the test-retest intra-observer CoR for LVM was 9 g (observer 1), 10.2 g (observer 2), 10.1 g (observer 3) and 12 g (observer 4).

For WB-MRA, of the 1488 arterial segments evaluated (31 anatomical locations for 48 volunteers) there was evidence of vessel luminal narrowing or stenosis in 53 (3.6%) segments, involving *n* = 29 volunteers. Details of the arterial segments where disease was noted by consensus are highlighted in Table 2. Of the 53 segments where disease was detected, 34 cases were scored as grade 1 (minor stenosis 1–50%), five cases were scored as grade 2 (moderate stenosis 51–70%), 11 cases were scored as grade 3 (severe stenosis 70–99%), and three cases were scored as grade 4 (vessel occlusion). However, the vast majority of segments were considered radiologically normal.

The greatest frequency of luminal narrowing was noted at the coeliac axis, where a stenosis score of grade 1 or more was noted in 18 of the volunteers. This was followed by the abdominal aorta (10 cases), and the right and left iliac arteries (four cases each). At all other segments, luminal narrowing was only ever noted in two (or less) of the 48 volunteers.

Independent single assessments of all datasets by each radiologist observer resulted in identical scoring between all four radiologist observers in 1277 (85.8%) of the 1488 arterial segments evaluated. Of those not scored identically, clear consensus agreement between three out of four radiologist observers was recorded for 162 (10.9%) of the segments. Radiological opinion was divided at the remaining 49 segments, i.e., 3.3% of the total reviewed. Fleiss' kappa values are listed for each of the arterial segments under investigation as a measure of interobserver agreement (Table 2). Worst case agreement was found at the coeliac axis (*k* = 0.66) and best case agreement was found at the innominate and left popliteal arteries (*k* = 1.00).

Intra-observer repeatability (derived from four equal subsets of *n* = 12 volunteers) resulted in radiologist observers 1 and 2 achieving consistent scoring in 356 (95.7%) of 372 arterial segments. For radiologist observers 3 and 4, consistent scoring was achieved in 346 (93%) and 350 (94.1%) of the segments respectively.

The median SAS score (equation 1) by consensus was 0.8%, and ranged from 0% to 5.6%. When the scores were evaluated between observers (using the Kruskal–Wallis test), there was no significant difference detected between the means of those provided by each radiologist observer (*p* = 0.14). Further, a subset of *n* = 12 volunteers were evaluated by each radiologist observer for a second time and when the SAS scores were compared on a per-radiologist basis (using the Wilcoxon signed rank test), there was no significant difference between the means of the first and second assessments (*p* = 0.74, *p* = 0.64, *p* = 0.71, and *p* = 0.71 for radiologist observers 1–4, respectively).

## Discussion

In the present study, a hybrid cardiovascular clinical MRI protocol was implemented from existing clinical sequences in order to acquire CE-MRA images of the human vascular tree together with assessment of heart structure, function, and LGE within a single examination. The data analysis methods are repeatable and all techniques are available on commercial systems, which allows for use at other centres.

The CMR arm of the protocol was implemented using recent recommendations.^1^, ^26^ Mean values for EF and LVM achieved overlap with published normal ranges,^3^, ^27^ although the mean value for LVM reported here is at the lower end. This is likely to be due to the segmentation approach, where contentious areas at the basal end of the LV were omitted if the section contained <50% full-thickness myocardium. This approach is particularly important for studies where MRI is used to detect small changes in LVM over time or in response to intervention.^28^ For this work emphasis was placed on the consistency of the acquisition and analysis between observers, and not the reporting of the LVM index (relative to body surface area), as the objective was more to identify a robust and repeatable protocol.

There are certain limitations associated with the CMR work reported here. The segmentation repeatability was optimized by consensus choice of basal section inclusion between observers. This approach was taken since any study involving the assessment of LVM using CMR (e.g., a longitudinal investigation) would normally account for consistency within the section ranges included. Therefore, the consensus choice resulted in a CoR that was representative of boundary contour placements. Even with this additional step included, the interobserver repeatability of 19.1 g was still quite variable but this is very much consistent with findings described in detail elsewhere where “real world” LVM inconsistencies of typically 28% may be expected for a large cohort.^29^ It is an inescapable fact that variability between experienced observers does still arise, even with the use of careful rules for how to deal with papillary muscles and trabeculae. This is further borne out from our work where far better repeatability was established for intra-observer data (CoR range 9–12 g for LVM).

For WB-MRA, the two-injection “standard dose” approach was chosen for three reasons. Firstly, the use of two stations per injection allowed for more sensitive control of bolus timing, and therefore, reliable arterial phase images. A single-injection approach was considered, but it was felt that this would require the use of less widely available time-resolved MRA techniques in order to ensure arterial phase data across the entire arterial tree. Secondly, the use of a contrast agent “standard dose” approach has been previously described on a similar cohort,^17^ where a dose reduction strategy (10 ml for injection 1 followed by 15 ml for injection 2) was recommended based on improved SNR and CNR for WB-MRA. Finally, the 15 min delay window between the first and second injections of the present two-stage approach provided a useful time to acquire CMR LGE data. Although a standard single dose of contrast agent is not generally considered optimal for LGE work at 1.5 T, recent evidence suggests that the difference between single and double doses for myocardial infarct detection at 3 T may not be significant.^30^ With future refinement, it would be possible to streamline the examination further by performing the first stage of the WB-MRA acquisition initially, and then undertaking the CMR LV assessment in the time window between injections before completing the WB-MRA acquisition. Other approaches to WB-MRA are also available, for example, it would be possible to acquire the head station with the initial injection, and then run a standard peripheral MRA protocol to acquire abdomen, upper leg, and lower leg stations with the second injection. However, this approach was found to be a little more prone to venous contamination at the lower leg position, so the described two-injection two station method was adopted instead because it enabled the lower leg station to be reached more quickly.

For WB-MRA, a similar data analysis approach was adopted to those reported elsewhere.^21^, ^22^ Vessel segments were assessed by a pool of four radiologist observers and assigned a score (from 0–4) based on the condition of the artery at that segment. The vast majority of arterial sites were considered normal (Table 2), although the abdominal aorta and coeliac axis were more commonly associated with some degree of pathological change (affecting up to 37.5% of the cohort). All individuals included in the study were clinically asymptomatic of cardiovascular disease but with a BNP level above the gender-specific mean, suggesting a potential cardiovascular abnormality.^31^

Recent published work indicates that radiological scoring of stenoses at the coeliac axis can result in false-positive assessments with MRI.^32^ This limitation may have been borne out by the present work, although it is not a certainty as a reference standard was not implemented (catheter angiography is not readily applicable to whole-body imaging). Observed stenoses at the coeliac axis were felt to be in keeping with median arcuate ligament compression (i.e., not an atherosclerotic process) and as such renders the inclusion of this in the atheroma score as questionable. Further work on larger cohorts will be required in order to establish the MRI normal variation for these arteries before the significance of these data become apparent. Aside from this, the interobserver repeatability of the scoring system was very good. Radiological opinion was divided in only 3.3% of the 1488 cases and Fleiss kappa (Table 2) demonstrated consistent good-excellent agreement between observers. On an interobserver basis, consistent scoring was achieved in 93% or more of the vessel segments reviewed and no significant variations between or within observers were detected.

The development of a whole-body atheroma score based on the accumulation of individual vessel segment scores has been reported previously.^22^ This was adapted to define a standard atheroma score (SAS) that reflects the total atheroma burden (as a percentage) across an individual subject. By including the number of segments assessed, it is possible to account for situations where small numbers of segments may be non-interpretable. For the present study there was only one segment (out of 1488) that was deemed non-interpretable by the radiologist observers. However, in cases where patients with significant CVD are studied then the number of non-interpretable segments may rise and the SAS would become less meaningful as a result.

In conclusion, the present study reported a dual-faceted MRI protocol applied to a cohort of healthy volunteers with low to medium risk of CVD. The protocol provides a comprehensive cardiovascular MRI examination of WB-MRA together with cardiac structure, function, and myocardial LGE with a mean examination time of 51 min. All MRI sequences are commercially available, and the analysis methods are repeatable between single and multiple observers. The technique is potentially suitable for future radiological investigation of patients with systemic CVD, and may also enable the effectiveness of targeted treatments to be monitored via individual, longitudinal, or multi-centre investigations.

## Figures and Tables

**Table 1:** Imaging parameters for all sequences run within the combined cardiac MRI and whole-body MR angiography (WB-MRA) protocol.

Protocol section	1	1	2	2	3	3	4	4
Description	CINE	CINE	WB-MRA	WB-MRA	TI scout	PSIR	WB-MRA	WB-MRA
Location	Heart	Heart LV	Station 1	Station 4	Heart LV	Heart LV	Station 2	Station 3
Sequence	2D TFi	2D TFi	3D TFl	3D TFl	2D TFi	2D PSIR	3D TFl	3D TFl
Cardiac phases	25	25	–	–	Variable	–	–	–
ECG gating	Retro	Retro	–	–	Pro	Pro	–	–
Lines/segment	14	26	–	–	9	25	–	–
Orientation	4ch & 2ch	SA	Coronal	Coronal	SA	SA	Coronal	Coronal
TR/TE (ms)	3.37/1.48	3.37/1.48	2.68/1	2.61/0.96	3.11/1.39	5.21/1.99	2.6/0.96	3.47/1.21
FA (°)	>50	>50	19	22	35	20	16	37
FOV (mm)	>360	>360	360 × 500	360 × 500	>360	>360	344 × 500	344 × 500
Phase FOV (%)	84.4	84.4	71.9	68.8	81.3	75	68.8	71.9
Section thickness (mm)	6	6	1.1	1	8	6	1.3	1.4
Sections (*n*)	1	2	96	80	1	2	96	96
Matrix (pixels)	216 × 256	173 × 256	313 × 512	277 × 448	78 × 192	144 × 256	264 × 512	242 × 448
Voxel size (mm)	Variable	Variable	1.1 × 1.0 × 1.1	1.2 × 1.1 × 1.0	Variable	Variable	1.3 × 1.0 × 1.3	1.5 × 1.1 × 1.4
Parallel imaging	×2	×2	×3	×3	–	×2	×3	×3
K-space	Linear	Linear	Linear	3D centric	Centric	Linear	3D centric	3D centric
BW (Hz/pix)	930	930	700	700	965	287	700	740
Scan time (s)	<20	<20	18	14	<20	<20	14	16

LV, left ventricle; TFi, TrueFISP; TFL, TurboFLASH; PSIR, phase sensitive inversion recovery; Retro, retrospective; Pro, prospective; 4ch, four chamber; 2ch, two chamber; SA, short axis; TR, repetition time; TE, echo time; TI, inversion time; FA, flip angle; FOV, field of view; i-PAT, integrated parallel acquisition technique; BW, bandwidth.

**Table 2:** Number of abnormal arterial assessments within the cohort (from a possible *n* = 48 for each location) identified by a consensus of four radiologist observers with cardiovascular MRI experience.

Arterial segment	Grade 1	Grade 2	Grade 3	Grade 4	Total (%)	Fleiss' kappa
R int. carotid	0	0	0	0	0 (0%)	0.89
L int. carotid	2	0	0	0	2 (4.2%)	0.90
R vertebral	0	0	0	0	0 (0%)	0.97
L vertebral	0	0	0	0	0 (0%)	0.94
Aortic arch	0	0	0	0	0 (0%)	0.99
Innominate	0	0	0	0	0 (0%)	1
R com. carotid	0	0	0	0	0 (0%)	0.99
L com. carotid	1	0	0	0	1 (2.1%)	0.93
R subclavian	2	0	0	0	2 (4.2%)	0.88
L subclavian	0	0	0	0	0 (0%)	0.90
Thoracic aorta	1	0	0	0	1 (2.1%)	0.86
Abdominal aorta	10	0	0	0	10 (21%)	0.81
Coeliac trunk	6	5	6	1	18 (37.5%)	0.66
Sup. mesenteric	1	0	0	0	1 (2.1%)	0.88
Inf. mesenteric	0	0	1	0	1 (2.1%)	0.91
R renal	1	0	0	0	1 (2.1%)	0.93
L renal	0	0	0	0	0 (0%)	0.94
R iliac	4	0	0	0	4 (8.4%)	0.90
L iliac	3	0	1	0	4 (8.4%)	0.87
R femoral	1	0	0	0	1 (2.1%)	0.91
L femoral	2	0	0	0	2 (4.2%)	0.91
R profunda	0	0	0	0	0 (0%)	0.96
L profunda	0	0	0	0	0 (0%)	0.99
R popliteal	0	0	0	0	0 (0%)	0.97
L popliteal	0	0	0	0	0 (0%)	1
R ant. tibial	0	0	0	0	0 (0%)	0.93
L ant. tibial	0	0	0	0	0 (0%)	0.97
R peroneal	0	0	1	0	1 (2.1%)	0.96
L peroneal	0	0	2	0	2 (4.2%)	0.95
R post. tibial	0	0	0	1	1 (2.1%)	0.96
L post. tibial	0	0	0	1	1 (2.1%)	0.93

The frequency of disease was highest at the coeliac axis.

Interobserver agreement for each site is described by Fleiss' kappa statistic.

R, right; L, left; Int, internal; Com, common; Sup, superior; Inf, inferior; Ant, anterior; Post, posterior.
